# Raising concerns about the Sepsis-3 definitions

**DOI:** 10.1186/s13017-018-0165-6

**Published:** 2018-01-25

**Authors:** Massimo Sartelli, Yoram Kluger, Luca Ansaloni, Timothy C. Hardcastle, Jordi Rello, Richard R. Watkins, Matteo Bassetti, Eleni Giamarellou, Federico Coccolini, Fikri M. Abu-Zidan, Abdulrashid K. Adesunkanmi, Goran Augustin, Gian L. Baiocchi, Miklosh Bala, Oussema Baraket, Marcelo A. Beltran, Asri Che Jusoh, Zaza Demetrashvili, Belinda De Simone, Hamilton P. de Souza, Yunfeng Cui, R. Justin Davies, Sameer Dhingra, Jose J. Diaz, Salomone Di Saverio, Agron Dogjani, Mutasim M. Elmangory, Mushira A. Enani, Paula Ferrada, Gustavo P. Fraga, Sabrina Frattima, Wagih Ghnnam, Carlos A. Gomes, Souha S. Kanj, Aleksandar Karamarkovic, Jakub Kenig, Faryal Khamis, Vladimir Khokha, Kaoru Koike, Kenneth Y. Y. Kok, Arda Isik, Francesco M. Labricciosa, Rifat Latifi, Jae G. Lee, Andrey Litvin, Gustavo M. Machain, Ramiro Manzano-Nunez, Piotr Major, Sanjay Marwah, Michael McFarlane, Ziad A. Memish, Cristian Mesina, Ernest E. Moore, Frederick A. Moore, Noel Naidoo, Ionut Negoi, Richard Ofori-Asenso, Iyiade Olaoye, Carlos A. Ordoñez, Mouaqit Ouadii, Ciro Paolillo, Edoardo Picetti, Tadeja Pintar, Alfredo Ponce-de-Leon, Guntars Pupelis, Tarcisio Reis, Boris Sakakushev, Hossein Samadi Kafil, Norio Sato, Jay N. Shah, Boonying Siribumrungwong, Peep Talving, Cristian Tranà, Jan Ulrych, Kuo-Ching Yuan, Fausto Catena

**Affiliations:** 1 Department of Surgery, Macerata Hospital, Macerata, Italy; 2 Department of General Surgery, Division of Surgery, Rambam Health Care Campus, Haifa, Israel; 3 General Surgery Department, Bufalini Hospital, Cesena, Italy; 4 Trauma Service, Inkosi Albert Luthuli Central Hospital, Durban, South Africa; 5 Department of Clinical Research & Innovation in Pneumonia and Sepsis, Vall d’Hebron Institute of Research (VHIR), Barcelona, Spain; 6 Division of Infectious Diseases, Cleveland Clinic Akron General, Akron, OH USA; 7 Department of Medicine, Northeast Ohio Medical University, Rootstown, OH USA; 8 Infectious Diseases Division, Santa Maria Misericordia University Hospital, Udine, Italy; 9 6th Department of Internal Medicine, Hygeia General Hospital, Athens, Greece; 10 Department of Surgery, College of Medicine and Health Sciences, UAE University, Al-Ain, United Arab Emirates; 11 Department of Surgery, College of Health Sciences, Obafemi Awolowo University, Ile-Ife, Nigeria; 12 Department of Surgery, University Hospital Centre, Zagreb, Croatia; 13 Department of Clinical and Experimental Sciences, University of Brescia, Brescia, Italy; 14 Trauma and Acute Care Surgery Unit, Hadassah Hebrew University Medical Center, Jerusalem, Israel; 15 Department of Surgery, Bizerte Hospital, Bizerte, Tunisia; 16 Department of General Surgery, Hospital San Juan de Dios de La Serena, La Serena, Chile; 17 Department of General Surgery, Kuala Krai Hospital, Kelantan, Malaysia; 18 Department General Surgery, Kipshidze Central University Hospital, Tbilisi, Georgia; 19 Department of Digestive Surgery, Cannes Hospital, Cannes, France; 20 Department of Surgery, School of Medicine, Pontifícia Universidade Católica do Rio Grande do Sul (PUCRS), Porto Alegre, Brazil; 21 Department of Surgery, Tianjin Nankai Hospital, Nankai Clinical School of Medicine, Tianjin Medical University, Tianjin, China; 22 Colorectal Unit, Addenbrooke’s Hospital, Cambridge University Hospitals NHS Foundation Trust, Cambridge, UK; 23 School of Pharmacy, Faculty of Medical Sciences, The University of the West Indies, St. Augustine, Eric Williams Medical Sciences Complex, Uriah Butler Highway, Champ Fleurs, Trinidad and Tobago; 24 Division of Acute Care Surgery, Program in Trauma, R Adams Cowley Shock Trauma Center, University of Maryland, Baltimore, MD USA; 25 Department of Surgery, University Hospital of Trauma, Tirana, Albania; 26 Sudan National Public Health Laboratory, Federal Ministry of Health, Khartoum, Sudan; 27 Department of Medicine, Infectious Disease Division, King Fahad Medical City, Riyadh, Saudi Arabia; 28 Department of Surgery, Virginia Commonwealth University, Richmond, VA USA; 29 Division of Trauma Surgery, Department of Surgery, School of Medical Sciences, University of Campinas (Unicamp), Campinas, SP Brazil; 30 Istituto Clinico San Rocco di Franciacorta, Brescia, Italy; 31 Department of General Surgery, Mansoura Faculty of Medicine, Mansoura University, Mansoura, Egypt; 32 Department of Surgery, Hospital Universitário Terezinha de Jesus, Faculdade de Ciências Médicas e da Saúde de Juiz de Fora, Juiz de Fora, Brazil; 33 Division of Infectious Diseases, Department of Internal Medicine, American University of Beirut, Beirut, Lebanon; 34 Clinic for Emergency Surgery, Medical Faculty, University of Belgrade, Belgrade, Serbia; 35 Third Department of General Surgery, Jagiellonian University Medical College, Krakow, Poland; 36 Department of Internal Medicine, Royal Hospital, Muscat, Oman; 37 Department of Emergency Surgery, City Hospital, Mozyr, Belarus; 38 Department of Primary Care and Emergency Medicine, Graduate School of Medicine, Kyoto University, Kyoto, Japan; 39 Department of Surgery, The Brunei Cancer Centre, Jerudong Park, Jerudong, Brunei; 40 Department of General Surgery, Faculty of Medicine, Erzincan University, Erzincan, Turkey; 41 Global Alliance for Infections in Surgery, Porto, Portugal; 42 Department of Surgery, Division of Trauma, University of Arizona, Tucson, AZ USA; 43 Department of Surgery, College of Medicine, Yonsei University, Seoul, South Korea; 44 Surgical Disciplines, Immanuel Kant Baltic Federal University/Regional Clinical Hospital, Kaliningrad, Russian Federation; 45 Department of Surgery, Universidad Nacional de Asuncion, Asuncion, Paraguay; 46 Clinical Research Center, Fundacion Valle del Lili, Cali, Colombia; 47 2nd Department of General Surgery, Jagiellonian University Medical College, Krakow, Poland; 48 Department of Surgery, Post-Graduate Institute of Medical Sciences, Rohtak, India; 49 Department of Surgery, Radiology, University Hospital of the West Indies, Kingston, Jamaica; 50 Infectious Diseases Division, Department of Medicine, Prince Mohamed Bin Abdulaziz Hospital, Ministry of Health, Riyadh, Saudi Arabia; 51 Second Surgical Clinic, Emergency Hospital of Craiova, Craiova, Romania; 52 Department of Surgery, Denver Health Medical Center, University of Colorado, Denver, CO USA; 53 Department of Surgery, Division of Acute Care Surgery, and Center for Sepsis and Critical Illness Research, College of Medicine, University of Florida, Gainesville, FL USA; 54 Department of Surgery, University of KwaZulu-Natal, Durban, South Africa; 55 Department of Surgery, Emergency Hospital of Bucharest, Bucharest, Romania; 56 Research Unit, Health Policy Consult, Weija, Accra, Ghana; 57 Department of Surgery, University of Ilorin, Teaching Hospital, Ilorin, Nigeria; 58 Department of Surgery and Critical Care, Universidad del Valle, Fundación Valle del Lili, Cali, Colombia; 59 Department of Surgery, Hassan II University Hospital, Medical School of Fez, Sidi Mohamed Benabdellah University, Fez, Morocco; 60 Department of Emergency Medicine, Santa Maria Misericordia University Hospital, Udine, Italy; 61 Department of Anesthesia and Intensive Care, Azienda Ospedaliero-Universitaria Parma, Parma, Italy; 62 Department of Surgery, UMC Ljubljana, Ljubljana, Slovenia; 63 Laboratory of Clinical Microbiology, Department of Infectious Diseases, Instituto Nacional de Ciencias Médicas y Nutrición Salvador Zubirán, Mexico City, Mexico; 64 Department of General and Emergency Surgery, Riga East University Hospital “Gailezers”, Riga, Latvia; 65 Emergency Post-Operative Department, Otavio De Freitas Hospital, Recife, Brazil; 66 General Surgery Department, Medical University, University Hospital St George, Plovdiv, Bulgaria; 67 Drug Applied Research Center, Tabriz University of Medical Sciences, Tabriz, Iran; 68 Department of Aeromedical Services for Emergency and Trauma Care, Graduate School of Medicine, Ehime University, Ehime, Japan; 69 Department of Surgery, Patan Hospital, Patan Academy of Health Sciences Lalitpur, Kathmandu, Nepal; 70 Department of Surgery, Faculty of Medicine, Thammasat University Hospital, Thammasat University, Rangsit, Pathum Thani Thailand; 71 Department of Surgery, North Estonia Medical Center, Tallinn, Estonia; 72 First Department of Surgery, First Faculty of Medicine, Charles University in Prague, Prague, Czech Republic; 73 Department of Emergency and Critical Care Medicine, Taipei Medical University Hospital, Taipei, Taiwan; 74 Department of Emergency Surgery, Parma Maggiore Hospital, Parma, Italy; 75 Department of Surgery, Nelson R Mandela School of Clinical Medicine, Durban, South Africa; 76 College of Medicine, Alfaisal University, Riyadh, Saudi Arabia; 77 Osvaldo Cruz Hospital Recife, Recife, Brazil; 78 General University Hospital in Prague, Prague, Czech Republic

**Keywords:** Sepsis, Septic shock, Organ dysfunction, Infections

## Abstract

The Global Alliance for Infections in Surgery appreciates the great effort of the task force who derived and validated the Sepsis-3 definitions and considers the new definitions an important step forward in the evolution of our understanding of sepsis. Nevertheless, more than a year after their publication, we have a few concerns regarding the use of the Sepsis-3 definitions.

## Background

The definition of sepsis has shifted over time.

Systemic inflammatory response syndrome (SIRS), sepsis, severe sepsis, and septic shock were initially defined in 1991 by a consensus panel convened by the American College of Chest Physicians (ACCP) and the Society of Critical Care Medicine (SCCM) [1]. The conference defined sepsis as a systemic inflammatory response syndrome (SIRS) due to infection. “Severe” sepsis was defined as sepsis associated with organ dysfunction, hypoperfusion, or hypotension, and “septic shock” was defined as sepsis with arterial hypotension despite adequate fluid resuscitation (Appendix 1).

The definitions were revisited in 2001 during the International Sepsis Definitions Conference, which included members from the ACCP, the SCCM, the American Thoracic Society (ATS), the European Society of Intensive Care Medicine (ESICM), and the Surgical Infection Society (SIS). [2] The consensus retained the definitions of sepsis as SIRS due to infection (presumed or confirmed) and severe sepsis as sepsis associated with acute organ dysfunction. However, the new criteria defining SIRS were greatly expanded from the 1991 original, and organ dysfunction variables indicative of severe sepsis were also defined. This new set of sepsis criteria also changed the diagnostic requirement from “more than 1” of the original 4 to “some” of the expanded list (Appendix 2).

In February 2016, the Journal of the American Medical Association (JAMA) published a proposal for new definitions and criteria for sepsis, called Sepsis-3 [3], updating previous sepsis definitions [1, 2]. The new definitions were prepared by a task force appointed by the European Society of Intensive Care Medicine (ESICM) and the Society of Critical Care Medicine (SCCM) (Appendix 3). They were aimed at providing a standardized classification to facilitate clinical care, future research, and reporting.

In 2017, the Global Alliance for Infections in Surgery instituted an interdisciplinary task force of 76 experts from 50 different countries with different backgrounds to assess the clinical value of the Sepsis-3 definitions.

The Global Alliance for Infections in Surgery appreciates the great effort of the task force who derived and validated the Sepsis-3 definitions and considers the new definitions an important step forward in the evolution of our understanding of sepsis particularly in regard to what distinguishes sepsis from uncomplicated infection. Nevertheless, more than a year after their publication, we have a few concerns regarding the use of the Sepsis-3 definitions [3]. We hope our comments can encourage further discussion and debate on how to further optimize the definitions of the sepsis continuum.

## Sepsis-3 definitions

The process for revising the Sepsis-3 definitions of sepsis and septic shock was a 2-year-long process that involved several components [3]. Critical efforts in this process included a discussion of the concept of sepsis, identification of criteria alerting clinicians for the patient’s risk to develop sepsis, and the development of the criteria to identify septic shock.

The clinical criteria for sepsis were formulated by using a data-driven approach. Electronic health record data of 1.3 million encounters at 12 community and academic hospitals within the University of Pittsburgh Medical Center health system in southwestern Pennsylvania were studied, and among them, there were 148,907 patients with suspected infection. The power of the Sequential [sepsis-related] Organ Failure Assessment (SOFA) was equivalent to Logistic Organ Dysfunction Score (LODS) and higher than SIRS in predicting hospital mortality in the intensive care unit (ICU). The choice of the SOFA to measure organ dysfunction was due to its greater simplicity. The task force introduced the rapid bedside quick SOFA (qSOFA) tool for determined patients outside the ICU who likely develop sepsis from the retrospectively derived databases. Confirmatory analyses were performed by using data sets from the USA and Germany.

The clinical criteria for septic shock were formulated by using multiple methods: (a) a systematic literature review and meta-analysis of the criteria used in observational studies reporting sepsis epidemiology, (b) a Delphi process among the 19 members of the task force to achieve consensus on the new definitions, and (c) cohort studies to test variables identified by the Delphi process using the Surviving Sepsis Campaign registry, along with two other data sets from the USA.

Yet when they were published, these definitions, however, had not been prospectively validated in a generalizable population. Moreover, the patient data were all almost exclusively from adults in high-income countries and primarily contained information from patients in the USA and Germany, so the utility of these definitions in other geographic regions and particularly in settings with less resources is unknown.

In recent years, the SIRS criteria were criticized for being too non-specific. The current use of two or more SIRS criteria to identify sepsis was unanimously considered by the task force to be unhelpful.

However, the task force stated that, although SIRS was not helpful in identifying patients with organ dysfunction, non-specific SIRS criteria still might have utility in identifying patients having infection.

Sepsis is now defined as life-threatening organ dysfunction caused by a dysregulated host response to infection. The aim of this definition is to emphasize the primacy of the non-homeostatic host response to infection, the potential lethality, and the need for urgent recognition.

Organ dysfunction can be represented by an increase in the Sequential [sepsis-related] Organ Failure Assessment (SOFA) score of 2 points or more (Table 1) which is associated with an in-hospital mortality greater than 10%.

**Table 1:** SOFA score

PaO_2_/FiO_2_ (mmHg)	SOFA score
< 400	1
< 300	2
< 200 and mechanically ventilated	3
< 100 and mechanically ventilated	4
Glasgow coma scale	
13–14	1
10–12	2
6–9	3
< 6	4
Mean arterial pressure OR administration of vasopressors required	SOFA score
MAP < 70 mm/Hg	1
dop ≤ 5 or dob (any dose)	2
dop > 5 OR epi ≤ 0.1 OR nor ≤ 0.1	3
dop > 15 OR epi > 0.1 OR nor > 0.1	4
Bilirubin (mg/dl) [μmol/l]	
1.2–1.9 [> 20–32]	1
2.0–5.9 [33–101]	2
6.0–11.9 [102–204]	3
> 12.0 [> 204]	4
Platelets ×10^3^/μl	
< 150	1
< 100	2
< 50	3
< 20	4
Creatinine (mg/dl) [μmol/l] (or urine output)	
1.2–1.9 [110–170]	1
2.0–3.4 [171–299]	2
3.5–4.9 [300–440] (or < 500 ml/d)	3
> 5.0 [> 440] (or < 200 ml/d)	4

Septic shock is defined as a subset of sepsis and should be clinically identified by a vasopressor requirement to maintain a mean arterial pressure of 65 mmHg or greater and serum lactate level greater than 2 mmol/l (> 18 mg/dl) in the absence of hypovolemia. The term “severe sepsis” is now superfluous and has been removed from the current definitions.

The Sepsis-3 definitions suggest that patients with at least two of these three clinical variables may be prone for the poor outcome typical of sepsis: (1) low blood pressure (SBP ≤ 100 mmHg), (2) high respiratory rate (≥ 22 breaths per min), or (3) altered mentation (Glasgow coma scale < 15) (quick SOFA). It is supposed to be useful in out-of-hospital, emergency surgery (ED), or general hospital ward settings, and patients with positive qSOFA should be used to further investigate for organ dysfuntion. The qSOFA score should not be regarded as a diagnostic criterion for defining sepsis. Rather, it should be regarded as a warning for patients with suspected infection who are likely to have poor outcomes.

## Raising concerns about the Sepsis-3 definitions

Sepsis is a multifaceted host response to an infecting pathogen that may be significantly amplified by endogenous factors. If left untreated, it may lead to the functional impairment of one or more vital organs or systems [4].There are many well-known risk factors for the infections that most commonly precipitate organ dysfunction, including acquired immunodeficiency syndrome, chronic obstructive pulmonary disease, many cancers, the use of immunosuppressive agents, and advanced age [5]. Although big steps forward have been made, the pathophysiological mechanisms for organ dysfunction are not entirely known, but it has become apparent that infection triggers a much more complex, variable, and prolonged host response, involving early activation of both pro- and anti-inflammatory responses.

Sepsis has variable clinical presentations depending on the initial site of infection, the causative organism, the pattern of acute organ dysfunction, and the underlying health status of the patient [5].

There is general consensus that early recognition and timely treatment largely determine outcome of sepsis.

Since the first classification in 1991 [1], the definition of sepsis, severe sepsis, and septic shock, though imprecise, have provided the clinicians a useful framework for clinical management, stressing the need for early recognition [6], and when these criteria are followed by the application of the Surviving Sepsis Campaign recommendations, they have an impressive history of success in reducing the mortality of sepsis in several areas of the world [7].

Several studies demonstrated that sepsis-related mortality reduced steadily over the years. A meta-analysis reported a reduction of sepsis 28-day mortality rates from 46.9% during the period 1991–1995 to 29% during 2006–2009 [8]. In the USA, mortality due to severe sepsis decreased by 51% from 1988 to 2012 [9]. In Australia and New Zealand, an overall decrease of 16.7% in hospital sepsis mortality was reported between 2000 and 2012 (from 35 to 18.4%) [10]. However, high mortality rates are still reported in low- and middle-income countries [11].

Despite decades of sepsis research, no specific therapies for sepsis have emerged. Without specific therapies, management is based on control of the infection and organ support.

Early antibiotics, source control, and fluid resuscitation support of vital organ function are the cornerstones for the treatment of patients with sepsis [12].

Timing and adequacy of source control are the most important issues in the management of patients with complicated intra-abdominal infections (cIAIs) because inadequate and late operation may have a negative effect on outcome. Source control was considered an essential element in the management of patients with complicated intra-abdominal infections (cIAIs) and should be considered and performed early after the diagnosis is established in most if not all patients [13]. Sotto et al. in 2002 found in a retrospective study that time between diagnosis and operation was associated with mortality [14]. In this study, the period between diagnosis and surgery was predictive of death within 30 days after diagnosis of peritonitis, emphasizing the importance of prompt surgical treatment. In the CIAOW (complicated intra-abdominal infections worldwide) observational study including 1898 consecutive patients older than 18 years undergoing surgery or interventional drainage to address IAI, a delayed initial intervention was found to be an independent variable predictive of mortality. In this study, the overall mortality rate was 10.5% (199/1898) [15].

To enable early interventions being effective, the diagnosis must be made as early as possible and treatment must be started early. The ability to identify septic patients who are at high risk for subsequent organ dysfunction and mortality, starting from pre-hospital care and ED, is crucial since timely recognition and appropriate, effective treatment substantially improves survival. This highlights the need for all healthcare workers to be vigilant about sepsis, so that the diagnosis can be made as early as possible [16].

One consequence of the new definitions is elimination of the concept of sepsis without organ dysfunction. Although the task force considered that the new definitions may better reflect the current understanding of sepsis pathophysiology, the literal interpretation of “sepsis” as a problem only when life-threatening organ dysfunction appears may be of limited utility in identifying patients who may benefit from early intervention.

The Sepsis-3 definitions requiring the presence of organ dysfunction to define sepsis may hinder the awareness of the importance of early recognition and treatment of infections before organ dysfunction appears, de-emphasizing intervention at earlier stages when it is most treatable.

Ideally, patients at risk for sepsis should be identified before organ dysfunction is established. Therefore, it may be questionable if it is helpful to have a definition that recognizes a patient once organ dysfunction has occurred and the patient already needs intensive care.

An observational study conducted at 132 medical institutions worldwide over a 4-month study period enrolled 4533 patients to validate a predictive score for patients with intra-abdominal infections [17]. Data from the WSES cIAIs Score Study (WISS) showed that mortality was significantly affected by the previous sepsis definition. Mortality with no sepsis was 1.2%, with sepsis only 4.4%, with severe sepsis 27.8%, and with septic shock was 67.8%. Severity of illness and the inherent mortality risk escalated from no sepsis, through sepsis, severe sepsis, and septic shock.

The previous stratification of the severity and consequent mortality due to infection that progressed from no sepsis to sepsis (infection meeting the criteria for systemic inflammatory response syndrome or SIRS), to severe sepsis (sepsis with organ failure, arterial hypertension, and/or hypoperfusion), to septic shock (arterial hypotension refractory to adequate volume resuscitation) is now reduced to simple infection, sepsis (infection and organ dysfunction), and septic shock (arterial hypotension defined as the use of vasopressors and hyperlactatemia). The previous concept of severe sepsis corresponds now to the definition of sepsis in the Sepsis-3 criteria, although this correlation is not absolute because sepsis, according to the new criteria, can include very different conditions, such as organ failure without hypotension nor hyperlactatemia [7].

The Sepsis-3 definitions exclude patients with isolated hypotension from the definition of sepsis because they would have a SOFA score of 1. Moreover, lactate is not part of the SOFA score, even though it is well documented to be a sensitive marker of severity of illness in patients with infection.

The Sepsis-3 definitions recommend using an increase in the SOFA score of 2 or more points to represent organ dysfunction. The SOFA score is intended to be used in the ICU and, to a lesser extent, the ED. Outside the ICU, SOFA was found only as good as the previous SIRS criteria (AUROC = 0.79 vs. AUROC = 0.76). Moreover, it is a valuable predictor of unfavorable outcome. The SOFA score was proposed in 1996 by the Working Group on Sepsis-Related Problems of the European Society of Intensive Care Medicine [18] to objectively describe the degree of organ dysfunction over time and to evaluate morbidity in patients in the ICU with sepsis. It was demonstrated to be a good indicator of prognosis in critically ill patients during the first few days of ICU admission [19]. The use of the SOFA score in research is commonly performed and constitutes a routine component of data collection for clinical trials in ICUs. However, the SOFA score is not universally accessible, especially for PaO_2_, which would require an arterial blood gas measurement.

Recognizing these practical limitations, the task force described a simplified method termed the “quick SOFA” (qSOFA) score to facilitate easier identification of patients potentially at risk of dying outside of critical care settings [20]. This instrument, which had not been validated in clinical practice at the time of Sepsis-3 publication, comprises three clinical parameters that are easy to assess outside the ICU.

The prognostic accuracy for in-hospital mortality of qSOFA is an area of great debate. A recent multicenter prospective cohort study involving 879 patients with suspected infection treated in the ED reported that qSOFA may be better than previous criteria at predicting in-hospital mortality among patients with suspected infection [21]. Nevertheless, we calculated the positive predictive value (PPV) of qSOFA for in-hospital death in this study to be only 0.24. This indicates that only one-quarter of the patients who had a qSOFA equal or more than 2 died in the hospital.

A recent large retrospective cohort analysis of 184,875 patients in 182 Australian and New Zealand intensive care units (ICUs) found that SOFA score had superiority in prediction of in-hospital mortality and that SIRS criteria had a greater prognostic accuracy for in-hospital mortality than qSOFA score [22].

In an observational cohort study performed at one ED at an urban university teaching hospital in Norway [23], qSOFA failed to identify two thirds of the patients admitted to an ED with severe sepsis. Further, qSOFA failed to be a risk stratification tool as the sensitivity to predict 7-day and 30-day mortality was low. The sensitivity was poorer than the other warning scores already in use at the study site and the SIRS criteria.

In a study of 886 patients, Tusgul et al. showed that the qSOFA score, SIRS criteria, and sepsis definition have low identification sensitivity in selecting septic patients in the pre-hospital setting or upon arrival in the ED [24].

An important limitation of the new definitions is the poor sensitivity of the qSOFA scoring system. This leads to a high number of false negatives and, subsequently, to a delayed diagnosis in many patients, which likely excludes its use as a screening tool for early sepsis, the stage in which treatment is most effective.

In a recent analysis of three prospectively collected, observational cohorts of 7754 infected emergency department patients aged 18 years or older, Henning et al. [25] demonstrated that the mortality rate for patients with a qSOFA score greater than or equal to 2 was 14.2%, with a sensitivity of 52% and specificity of 86% to predict mortality. In comparison, the original SIRS-based Sepsis-2 definition had a mortality rate of 6.8%, a sensitivity of 83%, and a specificity of 50%. Both the Sepsis-2 and Sepsis-3 definitions stratified patients at risk for mortality, with differing performances. In terms of mortality prediction, the new definitions had improved specificity but had very low sensitivity.

Williams et al. prospectively studied 8871 consecutive patients who were admitted from the ED with presumed infection and compared the diagnostic accuracy of SIRS with qSOFA and Sepsis-2 with Sepsis-3 definitions for organ dysfunction [26]. SIRS was associated with increased risk of organ dysfunction and mortality in patients without organ dysfunction. SIRS and qSOFA showed similar discrimination for organ dysfunction. qSOFA was specific but poorly sensitive for organ dysfunction. Mortality for patients with organ dysfunction was similar for Sepsis-2 and Sepsis-3, although 29% of patients with Sepsis-3 organ dysfunction did not meet Sepsis-2 criteria. Increasing numbers of Sepsis-2 organ system dysfunctions were associated with greater mortality.

In another study in which 3346 patients with infection outside the ICU and 1058 patients with infection in the ICU were analyzed, qSOFA provided inadequate sensitivity for early risk assessment [27].

Peake et al. performed a post hoc analysis of 1591 adult patients presenting to the ED with early septic shock [28]. At baseline, 1139 patients had a qSOFA score of ≥ 2. In contrast, 1347 participants met the Sepsis-3 criteria for sepsis. Of these, 1010 participants had a qSOFA score of ≥ 2 and met the Sepsis-3 criteria for sepsis. A quarter of participants who met the new sepsis definitions did not fulfill the qSOFA screening criteria, potentially limiting its utility as a screening tool for sepsis in the ED.

Sepsis requires urgent recognition because delayed treatment increases mortality. To optimize the timing of therapy, a screening test should be as sensitive as possible. Thus, it is preferable to have a more sensitive test with lower false negative results in order to not miss cases of serious sepsis.

Although some patients with ongoing sepsis may not have elevated lactate levels at presentation or during their clinical course [29], lactate measurement is advised as an important component of the initial evaluation of patients with sepsis. Elevated lactate levels (even if > 4 mmol/l) are no longer part of organ dysfunction criteria to define organ failure.

In the new definition of septic shock, hyperlactatemia is a required component for septic shock, differently from Sepsis-1 and Sepsis-2 definitions in which just the presence of refractory hypotension to fluid loading was considered shock. Therefore, when lactate measurements are not available, the diagnosis of septic shock can be more challenging and patients with potential shock will be considered as having only sepsis.

## Discussion

Sepsis was previously defined as a systemic inflammatory response syndrome (SIRS) in a patient with an infection but now reflects a significantly greater degree of illness, characterized by organ dysfunction, de-emphasizing intervention at earlier stages when it is most treatable.

Definition of organ failure based on SOFA ≥ 2 may be accurate and safe in the ICU, although reliance on the SOFA score can make it more challenging to diagnose a patient with sepsis outside the ICU environment.

The SOFA score is not globally accessible and not well known by emergency or ward healthcare professionals, and its applicability is complex outside the ICU, as it might demand the calculation of SOFA for subsequent days to verify if the patients fulfill the strict criteria and require laboratory tests [30].

The qSOFA is a tool for risk stratification, and it seems necessary to look for options to improve its low sensitivity. Until then, SIRS will still be helpful as a screening tool in ED to identify patients with infections that will most likely benefit from earlier and more aggressive interventions.

Clinicians should keep in mind the difference between a screening tool and a risk-stratification tool. A screening tool aims to identify patients with a particular disease from a larger pool of patients. Once these patients are identified, a risk-stratification tool can be applied to determine their likelihood of meeting a particular outcome.

We probably still need a good screening tool to identify patients at risk of developing organ dysfunction. However, this is not addressed in Sepsis-3.

The Sepsis-3 authors concluded that “These updated definitions and clinical criteria should clarify long-used descriptors and facilitate earlier recognition and more timely management of patients with sepsis or at risk of developing it.” However, it seems to us that Sepsis3 definitions have the opposite outcome.

Sepsis 3 definitions exclude from the concept of sepsis those patients who are in transition from infection to sepsis and who have a SOFA score of 2 or more points.

Probably, a pre-sepsis scoring concept is missing [31] and should be mandatory.

Fever, tachypnea, tachycardia, and increased WBC count are consistent features of critical illnesses, including those induced by infection. Although the SIRS criteria have been criticized for their lack of specificity, since 1991, they have gained widespread acceptance among clinicians all over the world and are still used worldwide to recognize early sepsis in clinical practice.

Instead of replacing the SIRS score with the new qSOFA score to identify early patients with sepsis, why not use both of them together, taking advantage of the sensitivity of SIRS and the specificity of qSOFA?

The new definitions are based on a retrospective evaluation of large hospital databases from two countries (the USA and Germany).The majority of sources of infection were hospital patients in referral centers with respiratory and postoperative infections. The target reader is an intensive care unit (ICU) physician. Although these definitions are of help for research purposes, they may not be representative of the wider clinical community [32].

Major international differences exist in the prevalence of infections, types of infecting microorganisms, and mortality rates. EPIC II demonstrated significant differences in Eastern Europe as compared to Western Europe, in Australasia as compared to Asia, and in Latin America as compared to North America [33].

Early recognition of sepsis is a general principle of sepsis management and is very important in low- and middle-income countries where the priorities for improving the quality of care for critically ill patients are different. Documenting the burden of critical illness in low-resource settings is challenging [29]. In these settings, a triage system that quickly recognizes critically ill patients and transfers them immediately to an acute care unit is a vital component of the emergency services. The most important challenges in the management of sepsis in these areas are triage and pre-hospital diagnosis. It should be done by very sensitive and non-invasive methods outside the hospital setting.

As a consequence, any process of improving quality of sepsis care globally should focus on simple diagnostic criteria based on physical examination findings that can recognize patients needing critical care. In these settings, a feasible, low-cost method of rapidly identifying patients requiring critical care is crucial. Early warning system scores utilize physiological, easy-to-measure parameters, assessing physiological parameters such as systolic blood pressure, pulse rate, respiratory rate, temperature, oxygen saturations, and level of consciousness [34]. They are simple, non-invasive, and easy-to-repeat measurement bedside tools. Large multicenter trials will be needed to explore if these findings can be shared all over the world.

## Conclusions

The pathogenesis of sepsis involves a complex interaction between the host immune system and the infecting microorganisms. Sepsis describes a broad-based syndrome covering many infectious agents, affecting various sites in patients of differing age, gender, and comorbidity. Its clinical manifestations are highly variable and may lead to severe organ dysfunction and death. Despite remarkable advances in the management of patients with sepsis, its recognition and timely, appropriate treatment remain of utmost importance.

The Sepsis-3 definitions underline the concept of a dysregulated immune response resulting in potentially modifiable life-threatening organ dysfunction. However, they may fail in identifying patients with serious infections before organ dysfunction ensues.

Downplaying infections that do not meet the current Sepsis-3 criteria may hinder their identification, resulting in an unnecessary increase in both morbidity and mortality due to their inexorable progression in the following hours.

Clear definitions for sepsis and septic shock should guide clinicians both to support early recognition of at-risk patients and to facilitate an understanding of the global epidemiology of sepsis.

Sepsis is a burden for global health. Its global nature calls for a global response, both in the geographic sense and across the whole range of sectors involved.

In this paper, we have raised our concerns regarding the Sepsis-3 definitions. We believe that in order for sepsis definitions to be universally accepted, they should facilitate clinical care on a global scale.

On Friday, May 26th, 2017, the World Health Assembly and the World Health Organization made sepsis a global health priority, by adopting a resolution to improve, prevent, diagnose, and manage sepsis [35].

The Global Alliance for Infections in Surgery suggests that future revisions have a more global perspective and include a wider range of representatives and expertise. We also hope our comments can serve as a basis for future discussions on how to further improve the definitions of the sepsis continuum.

## Appendix 1

### Sepsis-1 definitions

*SIRS (systemic inflammatory response syndrome)*

Presence of more than 1 of 4 findings:

- Body temperature > 38.0 or < 36.0 °C
- Heart rate > 90 beats/min
- Tachypnea > 20 breaths/min or hyperventilation with PaCO2 < 32 mmHg
- White blood cell (WBC) count > 12,000 cells/mm3 or < 4000 cells/mm3

*Sepsis*

SIRS in the presence of a confirmed or suspected infection.

*Severe sepsis*

Sepsis associated with organ dysfunction, hypoperfusion, or hypotension,

*Septic shock*

Sepsis with arterial hypotension despite adequate fluid resuscitation.

## Appendix 2

### Sepsis-2 definitions

*Sepsis*

Infection documented or suspected and some of the following parameters:

General parameters

- Fever (core temperature > 38.3 °C)
- Hypothermia (core temperature < 36 °C)
- Heart rate 90 bpm or > 2 SD above the normal value for age
- Tachypnea: > 30 bpm
- Altered mental status, significant edema or positive fluid balance (> 20 ml/kg over 24 h)
- Hyperglycemia (plasma glucose > 110 mg/dl or 7.7 mM/l) in the absence of diabetes

Inflammatory parameters

- Leukocytosis (white blood cell count > 12,000/μl)
- Leukopenia (white blood cell count < 4000/μl)
- Normal white blood cell count > 10% immature forms
- Plasma C reactive protein > 2 SD above the normal value
- Plasma procalcitonin > 2 SD above the normal value

Hemodynamic parameters

- Arterial hypotension (systolic blood pressure < 9 0 mmHg, mean arterial pressure < 70)
- Or a systolic blood pressure decrease > 40 mmHg in adults or < 2 SD below normal for age
- Mixed venous oxygen saturation > 70%
- Cardiac index > 3.5 l min^− 1^ m^− 2^
- Organ dysfunction parameters
- Arterial hypoxemia (PaO_2_/FIO_2_ < 300)
- Acute oliguria (urine output > 0.5 ml/kg^− 1^ h^− 1^ or 45 mM/l for at least 2 h)
- Creatinine increase ≥ 0.5 mg/dl
- Coagulation abnormalities (international normalized ratio > 1.5 or activated partial thromboplastin time > 60 s)
- Ileus (absent bowel sounds)
- Thrombocytopenia (platelet count 4 mg/dl or 70 mmol/l)

Tissue perfusion parameters

- Hyperlactatemia (> 3 mmol/l)
- Decreased capillary refill or mottling

## Appendix 3

### Sepsis-3 definitions

*Sepsis*

Life-threatening organ dysfunction due to a dysregulated host response to infection.

Sepsis clinical criteria: organ dysfunction is defined as an increase of 2 points or more in the Sequential Organ Failure Assessment (SOFA) score.

Patients with suspected infection who are likely to have a prolonged ICU stay or to die in the hospital can be promptly identified at the bedside with qSOFA. Two or more of:

- Hypotension: SBP less than or equal to 100 mmHg
- Altered mental status (any GCS less than 15)
- Tachypnoea: RR greater than or equal to 22

*Septic shock*

Subset of sepsis in which underlying circulatory and cellular/metabolic abnormalities are profound enough to substantially increase mortality.

Septic shock clinical criteria: Sepsis and (despite adequate volume resuscitation) both of:

Persistent hypotension requiring vasopressors to maintain MAP greater than or equal to 65 mmHg, and lactate greater than or equal to 2 mmol/l.

## Abbreviations

ED: Emergency department
IAI: Intra-abdominal infection
ICU: Intensive care unit
SIRS: Systemic inflammatory response syndrome
SOFA: Sequential [sepsis-related] Organ Failure Assessment
